# Gender-Associated Factors on the Occurrence and Prevalence of Zero-Dose Children in Sub-Saharan Africa: A Critical Literature Review

**DOI:** 10.3390/tropicalmed10100286

**Published:** 2025-10-06

**Authors:** Godfrey Musuka, Enos Moyo, Patrick Gad Iradukunda, Pierre Gashema, Roda Madziva, Helena Herrera, Tapiwa Dhliwayo, Constantine Mutata, Noah Mataruse, Oscar Mano, Elliot Mbunge, Tafadzwa Dzinamarira

**Affiliations:** 1 International Initiative for Impact Evaluation, Harare P.O. Box 0002, Zimbabwe; 2 Department of Public Health Medicine, University of KwaZulu-Natal, Durban 4041, South Africa; moyoenos@yahoo.co.uk; 3 Policy Research Centre, Department of Research, Kigali 00000, Rwanda; 4 Rwanda Food and Drug Authority, Kigali P.O. Box 3243, Rwanda; 5 School of Sociology and Social Policy, University of Nottingham, Nottingham NG7 2RD, UK; 6 School of Medicine, Pharmacy and Biomedical Sciences, University of Portsmouth, Portsmouth PO1 2UP, UK; 7 Department of Community Medicine, Midlands State University, Gweru P.O. Box 9055, Zimbabwe; 8 UNICEF, 2150 Copenhagen, Denmark; 9 Department of Public Health, University of the Western Cape, Robert Sobukwe Road, Bellville 7535, South Africa; 10 Department of Applied Information Systems, University of Johannesburg, Johannesburg 2092, South Africa; 11 School of Health Systems and Public Health, University of Pretoria, Pretoria 0002, South Africa; 12 ICAP in Zimbabwe, Harare P.O. Box 263, Zimbabwe

**Keywords:** gender, immunisation, zero-dose, sub-Saharan Africa, zero dose

## Abstract

Background: Immunisation remains one of the most effective and cost-efficient public health interventions for preventing infectious diseases in children. Despite global progress, Sub-Saharan Africa (SSA) continues to face challenges in achieving equitable immunisation coverage. Gender-related disparities, rooted in sociocultural and structural inequalities, significantly influence the prevalence of zero-dose and under-immunised children in the region. This review critically examines the gender-associated barriers to routine childhood immunisation in SSA to inform more inclusive and equitable health interventions. Methods: A critical literature review was conducted generally following some steps of the PRISMA-P and CRD guidelines. Using the Population–Concept–Context (PCC) framework, studies were selected that examined gender-related barriers to routine immunisation for children under five in Sub-Saharan Africa. Comprehensive searches were performed across PubMed, Google Scholar, and relevant organisational websites, targeting articles published between 2015 and 2025. A total of 3683 articles were retrieved, with 24 studies ultimately meeting the inclusion criteria. Thematic analysis was used to synthesise the findings. Results: Four major themes emerged: (1) women’s empowerment and autonomy, including limited decision-making power, financial control, and the impact of gender-based violence; (2) male involvement and prevailing gender norms, where patriarchal structures and low male engagement negatively influenced vaccine uptake; (3) socioeconomic and structural barriers, such as poverty, geographic inaccessibility, maternal workload, and service availability; and (4) education, awareness, and health system responsiveness. Conclusions: Gender dynamics have a significant impact on childhood immunisation outcomes in Sub-Saharan Africa. Future policies must integrate these insights to improve immunisation equity and reduce preventable child morbidity and mortality across the region.

## 1. Introduction

Immunisation is one of the most effective and cost-effective public health interventions for preventing infectious diseases in children [1]. Widespread immunisation coverage has significantly reduced morbidity and mortality from vaccine-preventable diseases (VPDs) globally [2]. However, despite the undeniable benefits, achieving and maintaining high levels of immunisation coverage remains a challenge in many countries, particularly for specific populations [3,4]. The Africa region has the highest prevalence of under-vaccinated and unvaccinated children. For instance, approximately 12.7 million children were under-vaccinated in 2021, and 14.5 million did not receive a single dose, also called “zero-dose” children in 2023 [5]. Among other challenges, gender-related barriers significantly contribute to inequalities in immunisation coverage and low immunisation rates. These barriers include harmful gender norms, lack of access to education, unequal power dynamics, limited mobility of women, limited decision-making among women in households and gender-based violence [6]. Gender disparities in immunisation rates persist in many parts of the world [7], including Sub-Saharan Africa. These disparities can be attributed to a complex interplay of factors, with social norms, economic inequalities, and limited access to education and information often playing a significant role [8]. Previous studies have highlighted a range of gender-related factors that influence immunisation uptake, including maternal education, autonomy in healthcare decision-making, and access to healthcare services [9,10,11]. For example, research has shown that children of mothers with limited decision-making power or restricted mobility are less likely to understand vaccination schedules [9,12]. Additionally, entrenched gender roles often result in women bearing the sole responsibility for child health without corresponding support or access to resources, further exacerbating disparities [13].

Despite growing recognition of these challenges, much of the existing literature is fragmented or context-specific, lacking a comprehensive synthesis of evidence across Sub-Saharan Africa. This review seeks to fill that gap by critically consolidating the available evidence to better understand how gender dynamics influence immunisation access and uptake, ultimately informing gender-sensitive policies and interventions across the region. This critical literature review aims to identify and analyse significant gender-related barriers to routine immunisation service delivery for children in Sub-Saharan Africa.

## 2. Methods

### 2.1. Study Design

A critical literature review of the available literature was conducted. The review loosely adhered to the Preferred Reporting Items for Systematic Reviews and Meta-Analysis Protocols (PRISMA-P) guidelines [14] and the Centre for Reviews and Dissemination (CRD) guidance for undertaking systematic reviews in healthcare [15].

### 2.2. Research Question and Study Eligibility

The population–concept–context (PCC) framework (Table 1) was employed to establish the eligibility criteria for the review question, in accordance with recommendations from the Joanna Briggs Institute [16].

This review addresses the following research question: What are gender-related barriers to routine immunisation service delivery for children in Sub-Saharan Africa?

### 2.3. Literature Sources

A comprehensive search was conducted on PubMed, Google Scholar, Web of Science electronic databases, and the websites of the WHO and UNICEF for articles reporting on routine immunisation service delivery. The review focused on articles published within the last 10 years to provide the most current information.

### 2.4. Search Strategy

A comprehensive search strategy was co-developed and pilot-tested in collaboration with a senior health science librarian. The search terms included gender, immunisation, barriers, vaccine hesitancy, decision-making, and education. Cultural practices, social norms, economic factors, health equity, and child health. The strategy was tailored for each database to ensure the retrieval of the most relevant studies. A comprehensive search strategy, along with PubMed findings, is presented in Table 2. The search strategy includes Medical Subject Headings (MeSH) and text-word searches. Grey literature was identified by consulting targeted websites for UNICEF and WHO, and by consulting contact experts within those organisations. Similar screening strategies well employed to ensure relevance.

### 2.5. Title, Abstract, Full-Text Screening

Two independent reviewers screened titles, abstracts, and full-text articles for eligibility. Studies identified through database searches were exported to EndNote [17]. Duplicate articles were removed. Studies were then exported from EndNote to the Covidence systematic review management platform [18]. This review was informed by the Preferred Reporting Items for Systematic Reviews and Meta-Analyses (PRISMA) guidelines; however, we did not strictly adhere to all items. Specifically, the review did not include prospective protocol registration, we broadened the inclusion criteria to include grey literature and organisational reports, and risk of bias assessments were not conducted in a standardised PRISMA format. These deviations reflect the nature of this study as a critical literature review rather than a fully systematic review, but the use of PRISMA elements still provided transparency and structure in reporting.

### 2.6. Data Abstraction and Analysis

A standardised data extraction form was developed specifically for this review. The form captured relevant information from the identified studies to address the research question. It extracted data that included author(s), publication year, publication type, study location, study design, population, barriers identified, other key findings and conclusion.

The extracted data was then analysed thematically. This involved identifying recurring themes and patterns related to gender-related barriers to immunisation in Sub-Saharan Africa. Notes were taken to categorise and organise the findings according to the Population, Concept, and Context (PCC) framework. This approach facilitated a comprehensive understanding of how gender intersects with various factors to create barriers to immunisation services for children in Sub-Saharan Africa.

## 3. Results

**Figure 1:**
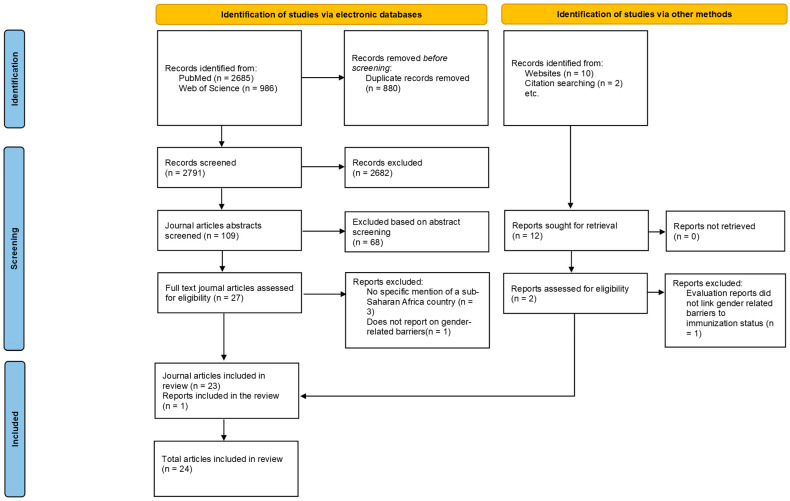
PRISMA flow diagram.

Our search retrieved 3683 articles. After removing duplicates, 2803 articles remained. Upon screening, 2682 articles were removed at the title screening stage, 78 at the abstract screening stage, and five at the full-text screening stage, leaving 24 for inclusion in this desk review (Figure 1).

### 3.1. Characteristics of Included Articles

Twenty-four articles were included [19,20,21,22,23,24,25,26,27,28,29,30,31,32,33,34,35,36,37,38,39,40,41,42]. Of these, two were reports [27,28]. The majority were cross-sectional studies, while seven employed qualitative methods [29,32,35,36,37,39,42]. More details are presented in Table 3.

### 3.2. Gender-Related Barriers to Routine Immunisation Service Delivery

Four themes emerged from the data analysis.

Theme 1: Women’s empowerment and autonomy

a. Decision-making power in health and household matters

Women’s ability to make autonomous decisions about their child’s health, especially immunisation, was a consistent predictor of vaccine uptake. In many households, male partners had final authority, limiting mothers’ access to services [23,25,27,36].

b. Financial resource access

Limited financial autonomy, including access to household income and decision-making around spending, was frequently cited as a barrier. Women who shared or had control over finances were more likely to vaccinate their children fully [24,31,37].

c. Influence of gender-based violence and social inequity

In contexts where women accepted or experienced gender-based violence or coercion, immunisation rates were lower [25]. Broader gender inequalities, such as restrictions on mobility or social participation, also undermined women’s health-seeking behaviours [40,42].

Theme 2: Male involvement and gender norms

a. Patriarchal decision structures and cultural norms

Deep-rooted social norms positioned men as decision-makers and excluded them from child health roles, often leading to active or passive resistance to immunisation [32,38,39].

b. Low male engagement in child health

Men were often disengaged from practical childcare tasks, such as attending health appointments. Where men participated, through providing support or transport, immunisation rates improved [26,35].

c. Stigma and gendered expectations

Women bore the burden of ensuring child health, and were blamed for missed vaccinations, even when decisions or barriers were beyond their control [36,37].

Theme 3: Socioeconomic Inequities and Structural Barriers

a. Poverty and occupational status

Economic disadvantage, particularly among single mothers, unemployed women, or those in casual work, limits access to immunisation services [19,30,32]. Feeling ashamed due to poor appearance or social status was also a deterrent [24].

b. Geographic and service accessibility

Rural women faced limited transport, long distances, and inadequate health infrastructure, further widening the urban-rural immunisation gap [19,28,40]. Similarly, mothers and children living in informal settlements encountered additional geographic and structural barriers. These communities often lacked nearby health facilities, faced overcrowding, and experienced irregular service delivery, reducing the consistency and reliability of immunisation access. Moreover, poor infrastructure, such as unpaved roads and insecure housing, created a physically and socially unstable environment that deprioritised preventive healthcare, including vaccination. Mobile populations, such as nomadic groups, faced even greater challenges. Their transient lifestyles often placed them outside the reach of static health systems, and a lack of proper identification or residence documentation further excluded them from immunisation registries and planning [28]. These geographical and structural exclusions compounded existing socioeconomic disadvantages, deepening immunisation inequities across both fixed and mobile underserved populations.

c. Maternal workload and time constraints

Heavy caregiving burdens, lack of childcare support, and household responsibilities constrained mothers’ time and energy to attend immunisation sessions [22,35].

Theme 4: Education, Awareness, and Health System Responsiveness

a. Maternal education and knowledge about vaccines

Mothers with no or low education levels were less likely to understand the benefits of immunisation and adhere to schedules [21,30,33].

b. Health Information access and media exposure

In many cases, women lacked access to information about immunisation due to limited literacy, media reach, or engagement with health extension services [26,40].

c. Gender-sensitive service design and ease of access to mothers

Several studies emphasised the lack of gender-sensitive planning in immunisation programmes. Health systems often failed to consider women’s needs, mobility restrictions, or potential for community mobilisation [35,41,42]. Further details are enclosed in Table 4.

## 4. Discussion

This review aimed to identify and synthesise gender-related factors influencing the access to and uptake of routine immunisation services for children in sub-Saharan Africa. The findings reveal that gender is an essential social determinant that intersects with socioeconomic, cultural, and structural barriers, shaping both the demand and supply of immunisation services. Our analysis highlights how gender norms and inequalities fundamentally influence caregivers’ ability to access and utilise these services, especially mothers.

### 4.1. Gendered Power Dynamics and Decision-Making Autonomy

One of the most consistent findings across studies was the role of women’s decision-making power in determining immunisation uptake [19,20,21,22,23,24,25]. Mothers with greater autonomy, especially regarding financial decisions and health-seeking behaviours, were significantly more likely to immunise their children fully. For instance, mothers who shared financial decision-making with partners had up to 8.1 times greater odds of fully vaccinating their children than those whose husbands retained sole control [21]. In contrast, male-dominated decision-making structures, which are usually shaped by patriarchal norms, often hinder timely vaccination. Even when women were willing to immunise their children, opposition or lack of support from male partners could result in missed vaccinations, illustrating how intra-household power dynamics serve as a gatekeeping mechanism that could act as barriers or facilitators to children’s health [22].

These findings underscore the importance of women’s empowerment as a critical enabler of child immunisation in issues of control over financial resources, health-related decision-making, and mobility. Where these domains are restricted, women’s capacity to prioritise or even access immunisation services is undermined. Notably, female-headed households, often considered more autonomous, had higher rates of under-immunisation, suggesting that autonomy alone is insufficient in contexts of poverty and weak health systems [25]. In addition to resource limitations, this review highlights the impact of existing attitudes and behavioural norms that may hinder vaccination uptake. This is where other factors uncovered by the critical literature review come to the fore.

### 4.2. Male Engagement and Gender Roles

From a delivery point of view, while much of the responsibility for child immunisation is placed on mothers, limited male engagement in child health creates a gendered burden that negatively affects immunisation uptake [20,24,35,37]. This can be due to sociocultural norms or a lack of awareness and initiative in the male parents and limited support provided to mothers, so they can access the point of delivery. Fathers were generally excluded from caregiving roles, and their involvement in vaccination was minimal. However, studies consistently show that when men are involved, vaccination rates improve significantly (AOR = 3.27, 95% CI: 1.84–5.81) [24]. Male involvement may look like providing transport, financial support for immunisation-related costs, accompanying mothers to clinics, supporting household chores, or just offering emotional and moral support besides their practical contributions. Fathers’ active support of vaccination can significantly influence the allocation of limited household resources, particularly in situations where conflicting demands may undermine preventive health initiatives.

This factor suggests that redefining caregiving from solely a woman’s responsibility to a shared parental role could improve immunisation outcomes.

Notably, male disengagement was not always passive. In some cases, opposition from fathers stemmed from misinformation, fear of vaccine side effects, or religious and cultural beliefs [36]. These findings highlight the urgent need for targeted health communication and outreach strategies that include men as partners in child health, challenge harmful gender norms, and promote shared decision-making. This is essential to empower not only women, but also men, to bring about change in this and the following generations on this critical public health intervention.

### 4.3. Maternal Education, Knowledge, and Age

Maternal education emerged as a robust predictor of immunisation, informed decision-making, financial independence, and social participation. Children of uneducated or illiterate mothers had significantly lower odds of being fully immunised [17,19,28,31]. Education likely functions through multiple pathways to enhance knowledge of vaccine benefits, increase health literacy, and possibly moreover empower women to make informed decisions and overcome barriers to immunisation, of which they may find fewer than those from lower cultural levels. Similarly, mothers with better knowledge about immunisation schedules were more likely to ensure complete vaccination.

Younger maternal age (especially 18–25 years) was associated with incomplete immunisation, possibly reflecting limited experience, lower autonomy, or constrained access to support systems [28]. Conversely, maternal age above 40 years was positively associated with full immunisation (AOR = 7.37, CI: 1.65–32), suggesting that older caregivers may possess greater confidence, autonomy, or social capital [24]. Further unknown factors may be influencing this group, despite similar confounders, such as financial resources and male support, to those of older women for the same outcomes.

### 4.4. Health System Responsiveness and Service Design

Gender-blind service delivery further perpetuated barriers. Immunisation services often failed to accommodate the unique needs of women, such as childcare responsibilities, mobility restrictions, or sociocultural expectations. This may be particularly true for younger women who have additional support needs. The health system’s limited outreach to male partners and lack of integration of gender-responsive strategies left many families navigating services within the constraints of unequal household dynamics. However, several studies noted that facility-based delivery, antenatal care (ANC) attendance, and postpartum home visits by health extension workers were associated with improved immunisation coverage. This emphasises the value of leveraging maternal health touchpoints for vaccination education and follow-up [24].

Health worker-patient gender discordance, especially in conservative settings, emerged as a barrier, with some women reluctant to access services from male providers. Moreover, poor media reach and inadequate communication channels resulted in women lacking key information about immunisation, especially in marginalised or low-literacy communities [40].

### 4.5. Socioeconomic and Structural Inequities

Gender-related barriers to immunisation were shown to be strongly influenced by structural determinants of health such as poverty, education, and geographic location. Economic disadvantage, particularly among women in low-income households, single mothers, or those engaged in informal work, limits access to services [17,28,29,30]. Some mothers expressed shame related to poverty, for example, being unable to dress appropriately for clinic visits, which further discouraged service utilisation [22]. Wealth disparities, combined with gendered financial dependency, meant that poorer women often lacked the resources to prioritise immunisation even when they were motivated to do so.

Geographic inequalities further complicated access. Women in rural areas faced long travel distances, limited transport options, and inadequate health infrastructure [17,26]. These barriers were particularly acute for mothers with childcare and domestic burdens, highlighting how the intersection of gender roles and spatial marginalisation creates compounding disadvantages. While improvements in rural coverage have been observed, the urban-rural gap remains a significant challenge in achieving equitable immunisation.

### 4.6. Cultural and Social Norms

Traditional gender norms were found to be pervasive, for instance, expectations that women alone are responsible for child health delivery [38,39,40] but not necessarily for broader decision-making with regard to prioritising and resourcing how and if healthcare should be delivered. These norms simultaneously placed the burden of immunisation on mothers while restricting their ability to fulfil it. Mothers were often blamed when children fell ill due to missed vaccinations, even when underlying causes included poverty, lack of partner support, or systemic barriers that were outside her control. Religious and cultural beliefs also played a role, with some communities using these ideologies to justify restrictions on women’s mobility or decision-making power within a culture of victim blaming, as women often suffer the consequences of poor child health.

Fear of vaccine side effects, especially among mothers who lacked spousal support, contributed to vaccine hesitancy [36,37]. When children experienced adverse reactions, women feared blame or violence, further reinforcing avoidance. These findings reveal a climate in which social expectations, emotional pressure, and limited autonomy converge to produce inequitable immunisation outcomes. Supporting these women in educating the next generation of children so that women can feel empowered and male carers understand the need for an equitable role can have a substantial impact on changing prevalent cultural and social norms.

## 5. Recommendations

Improving immunisation coverage in sub-Saharan Africa requires a gender-responsive approach that tackles the barriers faced by women, promotes shared responsibility for child health, and includes tailored education.

First, women’s decision-making empowerment must be addressed comprehensively, with a priority on maternal education. Education is a vital enabler of self-confidence and informed decision-making, fostering financial literacy and autonomy, which in turn leads to greater access to accurate health information. This will collectively enable more mothers to seek and complete immunisation for their children. This should be expanded beyond health education to encompass aspects that support the breaking of obstructive social norms, such as the fear of discrimination by healthcare providers or blame for adverse vaccination events. Programmes should support women through transport subsidies, flexible clinic hours, and integration of immunisation with maternal health services such as ANC and facility deliveries. They also show how the financial impact of vaccination can prevent the disease from posing a further burden on the family’s resources.

Second, male engagement is essential. Immunisation should be reframed as a shared parental responsibility. Involving men through targeted outreach, couple-based education, and community dialogues can reduce opposition and improve support for mothers. This could also widen their perceived responsibility concerning the day-to-day provision of healthcare to their family.

Third, health systems must recognise and respond to the realities of gender roles, with a focus on rural areas where religious and cultural norms have a stronger hold that limits the effectiveness of empowerment interventions. This includes addressing heavy maternal workloads, lack of male partner support, and religious and cultural restrictions on women’s mobility and autonomy. Gender-sensitive service delivery, outreach in hard-to-reach areas, and respectful interactions with providers are key. This should be particularly sensitive to vulnerable groups, such as single mothers or those with previous negative experiences when engaging with healthcare services.

Finally, policies and programmes must be informed by gender analysis and data. Collecting and using sex-disaggregated data, monitoring gender-related disparities, and designing interventions with a gender lens will help ensure equity and reach for all children, especially the zero-dose and under-immunised. This will be done while building a robust evidence base that can be used to rationalise resources and maximise the outcomes of investments in healthcare interventions.

## 6. Strengths and Limitations of the Review

The key strength of our study is the rigorous systematic methodology, as evidenced by the inclusion of diverse studies from across sub-Saharan Africa, focusing on an under-researched topic: the gendered dimension of immunisation.

The reviewed studies revealed several methodological limitations that may have influenced our findings. A significant issue was the reliance on convenience sampling, which often led to unrepresentative study populations, a lack of standard error measures, and selection bias. An additional limitation is that only articles published in English were considered, which might have introduced a language bias. Some of the studies included in this review were retrospective, which may have led to some findings being affected by recall bias. The inclusion of cross-sectional studies means that causality cannot be established with certainty.

Other limitations of our study include the limited availability of sex-disaggregated and zero-dose-specific data in some studies, the absence of critical appraisal of included studies, the lack of quantitative synthesis, and limited homogeneity in study designs, review quality, and comparability.

Additionally, the review process is susceptible to a potential selective reporting bias, as it relies heavily on the authors’ interpretations. Nonetheless, the findings provide insights critical to immunisation programmes in the region.

## 7. Conclusions

The review has highlighted the critical role of gender in shaping access to and uptake of childhood immunisation in sub-Saharan Africa. The persistence of zero-dose and under-immunised children is closely linked to gendered barriers ranging from limited maternal autonomy and male disengagement to poverty, poor service design, and restrictive social norms. Strategies to address these issues should not only involve those that seek to empower women but also engage men, reform health systems, and apply an intersectional gender lens to policy and programming. A more equitable and inclusive approach is essential to reach all children, particularly those who have been historically excluded, such as those residing in rural areas, from life-saving immunisation services.

## Figures and Tables

**Table 1:** Population–concept–context framework for this review.

Criteria	Determinants
Population	Children (0–5 years) and their caregivers (with a focus on gender-related influences)
Concept	Gender-related barriers to uptake and access to routine immunisation services
Context	Sub-Saharan Africa

**Table 2:** Search strategy and number of articles retrieved.

Search Strategy	Number of Articles Retrieved
Gender-related[All Fields] AND barriers[All Fields] OR (“vaccination hesitancy”[MeSH Terms] OR (“vaccination”[All Fields] AND “hesitancy”[All Fields]) OR “vaccination hesitancy”[All Fields] OR (“vaccine”[All Fields] AND “hesitancy”[All Fields]) OR “vaccine hesitancy”[All Fields]) OR (“decision making”[MeSH Terms] OR (“decision”[All Fields] AND “making”[All Fields]) OR “decision making”[All Fields]) OR (“education”[Subheading] OR “education”[All Fields] OR “educational status”[MeSH Terms] OR (“educational”[All Fields] AND “status”[All Fields]) OR “educational status”[All Fields] OR “education”[MeSH Terms]) OR “cultural”[All Fields]) AND practices[All Fields]) OR (“social norms”[MeSH Terms] OR (“social”[All Fields] AND “norms”[All Fields]) OR “social norms”[All Fields]) OR (“economic factors”[MeSH Terms] OR (“economic”[All Fields] AND “factors”[All Fields]) OR “economic factors”[All Fields]) OR (“health equity”[MeSH Terms] OR (“health”[All Fields] AND “equity”[All Fields]) OR “health equity”[All Fields]) OR (“child health”[MeSH Terms] OR (“child”[All Fields] AND “health”[All Fields]) OR “child health”[All Fields]) AND routine[All Fields] AND (“immunisation”[All Fields] OR “vaccination”[MeSH Terms] OR “vaccination”[All Fields] OR “immunisation”[All Fields] OR “immunisation”[MeSH Terms]) AND (“2015/05/21”[PDat]: “2025/05/21”[PDat])	2685 (PubMed), 986 Google Scholar

**Table 3:** Characteristics of included articles.

First Author, Publication Year	Reference	Country/Region	Publication Type	Study Design	Study Population
Yibeltal K, 2019	[19]	Ethiopia	Journal article	Demographic and Health Survey	Mothers with live children aged 12–23 months
Asabu MD, 2022	[20]	Ethiopia	Journal article	Systematic review and meta-analysis	Not specified
Desalew A, 2020	[21]	Ethiopia	Journal article	Systematic review and meta-analysis	Children aged 12–23 months
Tilahun B, 2020	[22]	Ethiopia	Journal article	Scoping review and Delphi method	Not specified
Ebot JO, 2015	[23]	Ethiopia	Journal article	Demographic and health survey	Children aged 12–30 months of married women aged 15–49 years
Bangura JB, 2020	[24]	Sub-Saharan Africa	Journal article	Systematic review	Not specified
Amoah A, 2023	[25]	Sub-Saharan Africa	Journal article	Demographic and health survey	Children aged 12–23 months
Gelagay AA, 2021	[26]	Ethiopia	Journal article	Cross-sectional study	Mothers of children aged 12–23 months
Project HOPE, Ministry of Health (Ethiopia), and Amref Health, 2022	[27]	Ethiopia	Report	Mixed methods	Children aged 12–35 months
UNICEF, 2018	[28]	Ethiopia	Report	Demographic and health survey	Children aged 12–23 months
Adeyanju GC, 2022	[29]	Malawi	Journal article	Qualitative	Information was obtained from caregivers, community and religious leaders, leaders of civil society groups, and teachers in schools
Dheresa M, 2021	[30]	Ethiopia	Journal article	Longitudinal	Children aged 12–24 months
Porth JM, 2021	[31]	Kenya	Journal article	Cross-sectional study	Women aged 15–49 years, currently married or living with a partner, had a living child aged 12–23 months
Jelle M, 2023	[32]	Somalia	Journal article	Qualitative	Female caregivers and purposively sampled nine vaccination service providers and six policy makers for interview
Abdallah MS, 2024	[33]	Sudan	Journal article	Cross-sectional	Parents of children aged 6–35 months
Lu X, 2021	[34]	Democratic Republic of Congo	Journal article	Cross-sectional	Women with children aged 12–23 months
Shearer JC, 2023	[35]	Democratic Republic of Congo, Mozambique and Nigeria	Journal article	Qualitative	Mothers of zero-dose and under-vaccinated children in selected communities
Abad N, 2017	[36]	Nigeria	Journal article	Qualitative	Administrative personnel, healthcare workers, caregivers, and community influencers
Gichuki, J 2024	[37]	Kenya	Journal article	Qualitative	Caregivers of children under five years of age residing in informal settlements
Nabwana BW, 2019	[38]	Uganda	Journal article	Cross-sectional	Caregivers of children under five years of age
Biks GA, 2024	[39]	Ethiopia	Journal article	Qualitative	Key informants from national and regional health authorities, multilateral and NGO partners, pharmaceutical supply services, and community-level stakeholders including leaders, Health Development Army members, and caregivers
Tekeba B, 2025	[40]	Ghana	Journal article	Cross-sectional	Children aged 12–25 months
Ngo-Bebe D, 2025	[41]	Democratic Republic of Congo	Journal article	Cross-sectional	Children aged 12–23 months
Etim EOE, 2025	[42]	Nigeria	Journal article	Qualitative	Mothers and community influencers who collaborate with healthcare workers to improve immunisation rates

**Table 4:** Findings from the included articles.

First Author, Year	Reference	Gender-Related Barriers
Yibeltal K, 2019	[19]	Wealth Inequality: The study found a significant gap in immunisation coverage between wealth quintiles. The poorest households consistently lag, indicating that economic disparities play a role in access to immunisation services. Women also often have less control over household finances, impacting their ability to prioritise immunisation. costs. Educational Disparities: Children of uneducated mothers were found to have the lowest immunisation coverage. This suggests that maternal education level influences access to and utilisation of immunisation services, indicating a gender-related barrier, as women often bear the primary responsibility for childcare. Urban-Rural Disparities: Immunisation coverage was consistently higher in urban areas compared to rural areas. Although improvements were observed in rural areas, the rural-urban inequality gap remains significant. This suggests that geographic location is a barrier, with rural populations facing challenges in accessing immunisation services. Limited transportation options in rural areas, especially for women with childcare responsibilities, can hinder reaching vaccination sites.
Asabu MD, 2022	[20]	Child gender: Child gender was investigated as a factor affecting access to immunisation. There was no evidence of any gender-based discrimination in childhood immunisation in Ethiopia.
Desalew A, 2020	[21]	Maternal education: Lower maternal education is linked to a higher likelihood of incomplete vaccination. Educated mothers are likely to be more aware of the benefits of vaccination schedules. Maternal knowledge: Women with a better understanding of vaccines are more likely to ensure their children are fully immunised. Maternal decision-making power: The study suggests that women with greater autonomy in decision-making are more likely to have their children fully vaccinated. In many contexts, women may not have the final say on healthcare decisions for their children. Place of delivery: Home births are associated with a higher risk of incomplete vaccination. Women delivering at health facilities receive counselling and reminders about vaccinations.
Tilahun B, 2020	[22]	Low decision-making power among mothers: The paper mentions mothers as primary caregivers for child immunisation, but also highlights a study that reveals mothers have limited decision-making power. In some cultures, fathers or other male figures may hold more authority regarding healthcare decisions for children. High workload on mothers: The paper discusses mothers’ heavy workload as a barrier to full immunisation. This could be because they are responsible for childcare and household duties, making it challenging to find time for vaccinations. Lack of support from male partners: The paper identifies a lack of support from male partners as a contributing factor. This implies that mothers might need male involvement or approval for taking children to be vaccinated.
Ebot JO, 2015	[23]	Financial decision-making power: Only decisions related to finances had a significant effect on partial immunisation status. In the first complete model, for example, women who reported that their husbands made the final decision on their earnings had 4.3 times higher odds of their children being partially immunised versus having no vaccines, net of all control variables. In the second complete model, joint decisions on earnings (compared to husbands making the sole earnings decision) increased the odds of children being fully immunised by 8.1 times versus not being vaccinated.
Bangura JB, 2020	[24]	Poverty: Feeling ashamed of poverty-associated reasons, e.g., Mothers who thought that they could not dress smartly enough for the approval of other women at the clinic were less likely to attend. Marital status: Being a single mother was also cited as a barrier to childhood immunisation. Decision-making process: The decision for immunisation was generally a joint decision between the child’s mother and father. But it was noted with strong emphasis that women were in charge of taking children for immunisation and sometimes the husbands opposed immunisation and stopped their wives from immunising their children by denying them the social and financial support necessary.
Amoah A, 2023	[25]	Gender-based violence: Children of mothers with higher acceptance toward violence were less likely to be fully immunised [aOR = 0.90, CI 0.81, 0.99]. Decision-Making Autonomy: The odds of full immunisation were higher among children born to mothers with high [aOR = 1.11, CI 1.01, 1.22] decision-making capacity.
Gelagay AA, 2021	[26]	Maternal Age: Mother age >40 years (AOR = 7.37, 95% CI: 1.65, 32) was positively associated with being fully vaccinated. Women’s empowerment: Mothers who initiate vaccine uptake (women’s empowerment) (AOR = 1.57, 95% CI: 1.13–2.39) was positively associated with being fully vaccinated. ANC attendance: Mothers who had 1–3 ANC visits (AOR = 2.51, 95% CI: 1.14, 5.52), and 4+ ANC follow-up were positively associated with being fully vaccinated (AOR = 2.73, 95% CI: 1.26, 5.91). Health extension worker visits: Health extension workers’ home visit during the first weeks of the postpartum period was positively associated with being fully vaccinated (AOR = 1.76, 95% CI: 1.10, 2.84). Involvement of the male partner: Males involved in child immunisation (AOR = 3.27, 95% CI: 1.84, 5.81) were positively associated with being fully vaccinated. Birth order: Birth order of 6 and above (AOR = 0.35, 95% CI: 0.14, 0.86) was negatively associated with being fully vaccinated.
Project HOPE, Ministry of Health (Ethiopia), and Amref Health, 2022	[27]	Decision-Making Autonomy: Female-headed households had higher rates of under-immunised children (72.0%) and dropout rates (56.0%). The prevalence of zero-dose and under-immunised children and dropout rates declined with women’s increasing power in household decision-making. Type of occupation: Vaccination rates did not show consistent differences across types of women’s occupation; however, women engaged in professional jobs had substantially better outcomes. Wealth index: Women’s land and house ownership are associated with lower rates of zero-dose, under-immunisation, and drop-out. Gender roles: Fathers’ support in household chores is associated with lower rates of zero-dose, under-immunisation, and drop-out. Financial decision-making power: Engagement of both partners in household resource allocation is associated with lower rates of zero-dose, under-immunisation, and drop-out. Access to information: Women’s better access to information about what is happening in the community is associated with lower rates of zero-dose, under-immunisation, and drop-out.
UNICEF Ethiopia, 2018	[28]	The main determinants associated with inequalities in coverage are the geographic area where the child lives, household wealth, caregivers’ education, and place of residence (urban vs. rural). There were no significant differences in vaccination coverage between boys and girls.
Adeyanju GC, 2022	[29]	Decision-making process: Husband influenced vaccination decision-making leading to the children being unvaccinated despite their mother’s willingness.
Dheresa M, 2021	[30]	Age: Young maternal age (18–25 years) is associated with increased odds of partial or no vaccination. Maternal age 25–33 years increases the odds of partial immunisation. Maternal age 34–42 years increases the odds of partial immunisation. Education: Uneducated mothers have higher odds of having non-vaccinated children. Mothers who cannot read or write are more likely to have non-vaccinated children. Employment status: Unemployed mothers have increased odds of their children being partially or not vaccinated compared to housewives.
Porth JM, 2021	[31]	Wealth status: Higher enabling conditions among middle-wealth women significantly increase the likelihood of having a fully vaccinated child. Middle level of empowerment among the wealthiest women is associated with a higher likelihood of full child vaccination.
Jelle M, 2023	[32]	Wealth status: Single mothers without livelihoods often engage in casual labour, which limits their ability to access vaccination services. Male-dominated decision-making: husbands make the final decision on child vaccination, and if they oppose it, children remain unvaccinated.
Abdallah MS, 2024	[33]	Mothers’ education: Mothers with primary education were more likely to partially vaccinate their children with the pentavalent vaccine, followed by those with secondary education, and then those with no education. Other: Parental perception of the importance of male vaccination was significantly associated with the vaccination status of children. Findings showed that about one in five parents perceived male vaccination as more important than female vaccination.
Lu X, 2021	[34]	Women’s empowerment: Children of women with high levels of empowerment had higher odds of complete vaccination, with values of 1.63 (*p* = 0.002) and 1.59 (*p* = 0.012) for intrinsic agency and enabling resources of the empowerment, respectively, compared to the children of women with low levels of empowerment.
Shearer JC, 2023	[35]	Most caregivers reported some difficulty juggling their gender-prescribed tasks related to childcare and domestic work with getting a child vaccinated. These difficulties were more common among caregivers who faced other financial or time-related resource barriers, whether because of poverty or because the child’s father worked or lived away from the home. Gender inequality was sometimes apparent in the caregivers’ lack of agency to decide whether to vaccinate their child. When husbands assisted with practical aspects, such as childcare or transportation, as reported by some respondents, caregivers were more likely to seek vaccination.
Abad N, 2017	[36]	Patriarchal decision-making: Men control healthcare decisions and can block access to immunisation. Female disempowerment: Women lack the agency to take their children for immunisation services. For example, men use religious beliefs to justify restricting women’s actions. Women lack the power or autonomy to challenge these beliefs or make independent decisions.
Gichuki J, 2024	[37]	Maternal burden of responsibility: Mothers are blamed for vaccine-preventable illness, yet lack full agency. Limited financial autonomy: Mothers often require male approval or support to cover transportation and fees. Male disengagement: Fathers are seen as supporters, not decision-makers, reinforcing gender roles. Cultural and emotional pressure: Women are expected to prioritise child health, often without adequate support.
Nabwana BW, 2019	[38]	Male opposition due to misinformed beliefs: Fathers stopped immunisation because of typical post-vaccine side effects (e.g., crying). Low male knowledge about immunisation: Fathers’ ignorance about the benefits of vaccines led to opposition. Patriarchal decision-making: Fathers’ decisions override mothers’ intentions to immunise their children.
Biks GA, 2024	[39]	Immunisation of children is seen as women’s responsibility: Men are socially excluded from child health roles. Lack of male engagement in immunisation: No shared decision-making or support; women face challenges alone. Fear of side effects among mothers: Mothers avoid vaccination to avoid blame or distress if their child experiences a reaction. Religious/cultural beliefs reinforce female-only roles: Past religious objections were voiced primarily by women, strengthening their sole responsibility.
Tekeba B, 2025	[40]	Low female autonomy in healthcare decisions: When women lack a say in health matters, children are less likely to be vaccinated. Limited access to maternal health services: Barriers to ANC and facility delivery, often rooted in gender norms, reduce vaccine uptake. Unequal access to health information: Women in low-media-exposure areas may not receive critical immunisation messages. Regional disparities linked to gender inequality: Lower coverage in some areas may reflect entrenched patriarchal norms limiting women’s agency.
Ngo-Bebe D, 2025	[41]	Sociocultural gender norms: Traditional gender roles limit women’s ability to access or promote immunisation. Lack of gender considerations in planning: Immunisation services were not initially designed with gender-specific barriers in mind. Women’s underutilised role in outreach: Before the intervention, women’s potential to mobilise communities was not fully tapped.
Etim EOE, 2025	[42]	Limited female decision-making: Women lack the autonomy to seek child immunisation independently. Health provider-patient gender discordance: Male-dominated healthcare workforce restricts women’s access in conservative regions. Restricted female mobility: Cultural norms limit women’s ability to travel alone or far from home. Social norms and religious beliefs: Gender roles discourage women from engaging in public or healthcare settings.

## Data Availability

The original contributions presented in this study are included in the article. Further inquiries can be directed to the corresponding author.
